# CD40-Activated B Cell Cancer Vaccine Improves Second Clinical Remission and Survival in Privately Owned Dogs with Non-Hodgkin's Lymphoma

**DOI:** 10.1371/journal.pone.0024167

**Published:** 2011-08-31

**Authors:** Karin U. Sorenmo, Erika Krick, Christina M. Coughlin, Beth Overley, Thomas P. Gregor, Robert H. Vonderheide, Nicola J. Mason

**Affiliations:** 1 Department of Clinical Studies, University of Pennsylvania, Philadelphia, Pennsylvania, United States of America; 2 Department of Pathobiology, School of Veterinary Medicine, University of Pennsylvania, Philadelphia, Pennsylvania, United States of America; 3 Department of Medicine, School of Medicine, Abramson Family Cancer Research Institute, University of Pennsylvania, Philadelphia, Pennsylvania, United States of America; 4 Abramson Cancer Center, University of Pennsylvania, Philadelphia, Pennsylvania, United States of America; National Cancer Institute, United States of America

## Abstract

Cell-based active immunotherapy for cancer is a promising novel strategy, with the first dendritic cell (DC) vaccine achieving regulatory approval for clinical use last year. Manufacturing remains arduous, especially for DC vaccines, and the prospect of using cell-based immunotherapy in the adjuvant setting or in combination with chemotherapy remains largely untested. Here, we used a comparative oncology approach to test the safety and potential efficacy of tumor RNA-loaded, CD40-activated B cells in privately owned dogs presenting with non-Hodgkin's lymphoma (NHL), a clinical scenario that represents not only a major problem in veterinary medicine but also a bona fide spontaneous animal model for the human condition. When administered to NHL dogs in remission after induction chemotherapy, CD40-B cells electroporated *ex vivo* with autologous tumor RNA safely stimulated immunity *in vivo*. Although chemotherapy plus CD40-B vaccination did not improve time-to-progression or lymphoma-specific survival compared to dogs treated with chemotherapy alone, vaccination potentiated the effects of salvage therapy and improved the rate of durable second remissions as well as subsequent lymphoma-specific survival following salvage therapy. Several of these relapsed dogs are now long-term survivors and free of disease for more than a year. Overall, these clinical and immunological results suggest that cell-based CD40 cancer vaccination is safe and synergizes with chemotherapy to improve clinical outcome in canine NHL. More broadly, our findings underscore the unique value of clinical investigations in tumor-bearing companion animals.

## Introduction

With the recent FDA approval of the dendritic cell vaccine sipuleucel-T for the treatment of advanced prostate cancer [1], there has been renewed efforts to further optimize second-generation cell-based therapies aimed at stimulating anti-tumor immunity for the treatment of cancer [2]–[3]. Because the use of resting or unactivated antigen presenting cells (APC) may actually trigger antigen-specific T cell tolerance and therefore be highly counter-productive if administered to patients, it is critical to develop and test APC formulations that are expressly designed to activate APC either *in vivo* or *ex vivo*. One approach that has emerged is pharmacological crosslinking of CD40 expressed on the surface of APC [4] in order to up-regulate MHC and co-stimulatory molecules, stimulate secretion of inflammatory cytokines such as IL-12, and up-regulate anti-apoptotic molecules – a process collectively known as “APC licensing” that enables priming of naïve CD4+ and CD8+ T cells and boosting robust memory T cell responses [5]. Clinical reagents that activate CD40 have been developed for systemic use and have shown clinical promise [6]–[8], but these agents do not necessarily drive T cell responses *in vivo* owing to restrictive features of the tumor microenvironment [8]. Here, we study an *ex vivo* approach to APC licensing. Although dendritic cells in most models are the cells chiefly responsible for physiological T cell priming *in vivo*, B cells can also act as APC and can also be licensed by CD40 activation [9]–[10]. CD40-activated B cells (CD40-B), like DC, can prime naïve T cell responses against neoantigens *ex vivo* and can boost memory T cell responses [9]–[11], suggesting that antigen-loaded, CD40-B may represent a viable alternative to DC in cell-based vaccination strategies. In sharp contrast to DC, however, B cells can be readily expanded *in vitro* using CD40 ligation, removing the necessity of large volume leukapheresis (as is required to manufacture sipuleucel-T) to procure sufficient numbers of APC for recurrent vaccinations [9]–[10], [12]. Although CD40-B can augment antigen-specific effector T cell responses *in vitro* against viral and tumor associated antigens, little is known whether these alternative APC can stimulate effective anti-tumor immune responses *in vivo*.

To address this question, we have performed a clinical trial of a CD40-B vaccine in privately owned dogs (pets) with non-Hodgkin's lymphoma (NHL). Canine NHL represents a major health issue in dogs and shares similar clinical, behavioral, genetic and cytogenetic characteristics to NHL in humans. It is now widely accepted as a clinically relevant model system in which to evaluate safety and efficacy of novel therapeutic agents prior to their translation into human clinical trials [13]. As in human patients with NHL, the standard of care for the initial treatment of canine NHL consists of combination chemotherapy protocols. Although induction chemotherapy is highly effective and 60%–85% of treated dogs achieve complete clinical remission following induction therapy, nearly all dogs relapse within one year with drug-resistant disease and die from lymphoma [14]–[15]. The cure rates of canine NHL have not changed substantially over the past decades despite the use of more dose-intense protocols.

 In this study, we clinically tested a therapeutic platform that we have previously described in preclinical studies [12] in which B cells from NHL dogs are expanded *in vitro* using CD40L transfected K562 cells (KtCD40L) and then loaded with autologous tumor RNA to generate a cell-based vaccine. RNA-loaded CD40-B derived from canine peripheral blood B cells stimulate functional, antigen-specific T cell responses *in vitro* from healthy dogs and from dogs with spontaneously occurring NHL [12]. The use of whole tumor RNA as the antigenic payload allows for an HLA-independent, whole antigen approach that aims to promote a polyclonal anti-tumor T cell response. Here, we report the first clinical trial in any species of a tumor RNA loaded CD40-B cancer vaccine, performed in the setting of minimal residual disease. Our results demonstrate that CD40-B can stimulate immune responses *in vivo* that impact second remission and survival in a spontaneous, large animal model of NHL. These results suggest that tumor antigen loaded CD40-B may serve as a practical alternative to DC in cell-based vaccine strategies for both dogs and humans with cancer.

## Results

### Privately owned dog protocol

Thirty privately owned dogs with NHL were enrolled at disease presentation in this pre-clinical feasibility trial and underwent induction chemotherapy (Group 1; intent to treat). Patient characteristics are shown in Table 1. Nineteen dogs (63%) were confirmed to be in complete cytological and clinical remission at the end of chemotherapy and were therefore eligible for vaccination with autologous CD40-activated B cells (every other week for three intradermal injections in the flank) that had been loaded with total RNA previously isolated from autologous lymphoma cells (Methods and Materials, and Supplemental Methods) (Group 2). An equal number of autologous CD40-B loaded with canine distemper virus hemagglutinin mRNA was injected in the opposite flank as an immunological control. Sixty-four dogs with NHL were selected from a group of 130 dogs as controls for the unvaccinated group (Group 3). These dogs were selected based on the fact that they were treated with the same induction chemotherapy regimen as the vaccinated group (Group 2) and were confirmed to be in complete clinical and cytological remission at the end of the chemotherapy protocol but they did not receive CD40-B cell vaccines. There were no incentives for dogs in any group to receive chemotherapy thus avoiding any bias in treatment approach or intensity of therapy. Although the number of dogs in the control group (Group 3) was larger than the vaccinated dog group (Group 2), there were no statistically significant differences in prognostic factors including immunophenotype between groups 2 and 3 (Table 1).

**Table 1:** Canine patient characteristics.

Variable	Group 1 (intent to treat) (n = 31)	Group 2 (vaccinated) (n = 19)	Group 3 (control) (n = 64)	P-value (Group 2 vs. 3)
Age (yrs; mean ± SD)	8.03 (2.7)	7.6 (2.7)	7.33 (2.7)	0.73
Body weight (kg; mean ± SD)	32.9 (13.5)	33.1 (12.7)	32.3 (13.1)	0.82
Stage	n (%)	n (%)	n (%)	0.43
II–IV	17 (54.8%)	9 (47.4%)	27 (42.2%)	
V	14 (45.2%)	10 (52.6%)	37 (57.8%)	
Sub-stage	n (%)	n (%)	n (%)	0.112
a	21 (67.7%)	14 (73.7%)	35 (54.7%)	
b	10 (32.3%)	5 (26.3%)	29 (45.3%)	
Immunophenotype	n (%)	n (%)	n (%)	0.45
B	21 (67.7%)	14 (73.7%)	33 (78.6%)	
T	10 (32.3%)	5 (26.3%)	9 (21.4%)	

### Toxicity

Vaccination with RNA loaded CD40-B was extremely well-tolerated. One dog exhibited signs of an acute systemic reaction, including weakness, hypotension, and vomiting within a few hours of receiving the first vaccine. Treatment with intravenous fluids, diphenhydramine and anti-emetics resulted in an uneventful recovery. This particular dog was pretreated with diphenhydramine immediately prior to the 2 subsequent vaccinations, and no further systemic reactions occurred. Mild injection site reactions including erythema and swelling occurred in several dogs. There were no significant alterations in complete blood chemistries during or after vaccination in any of the dogs. Furthermore, no long-term complications of vaccination such as autoimmunity were identified clinically, clinicopathologically (monitored by serial CBCs) or at necropsy.

### Clinical outcomes

**Figure 1:**
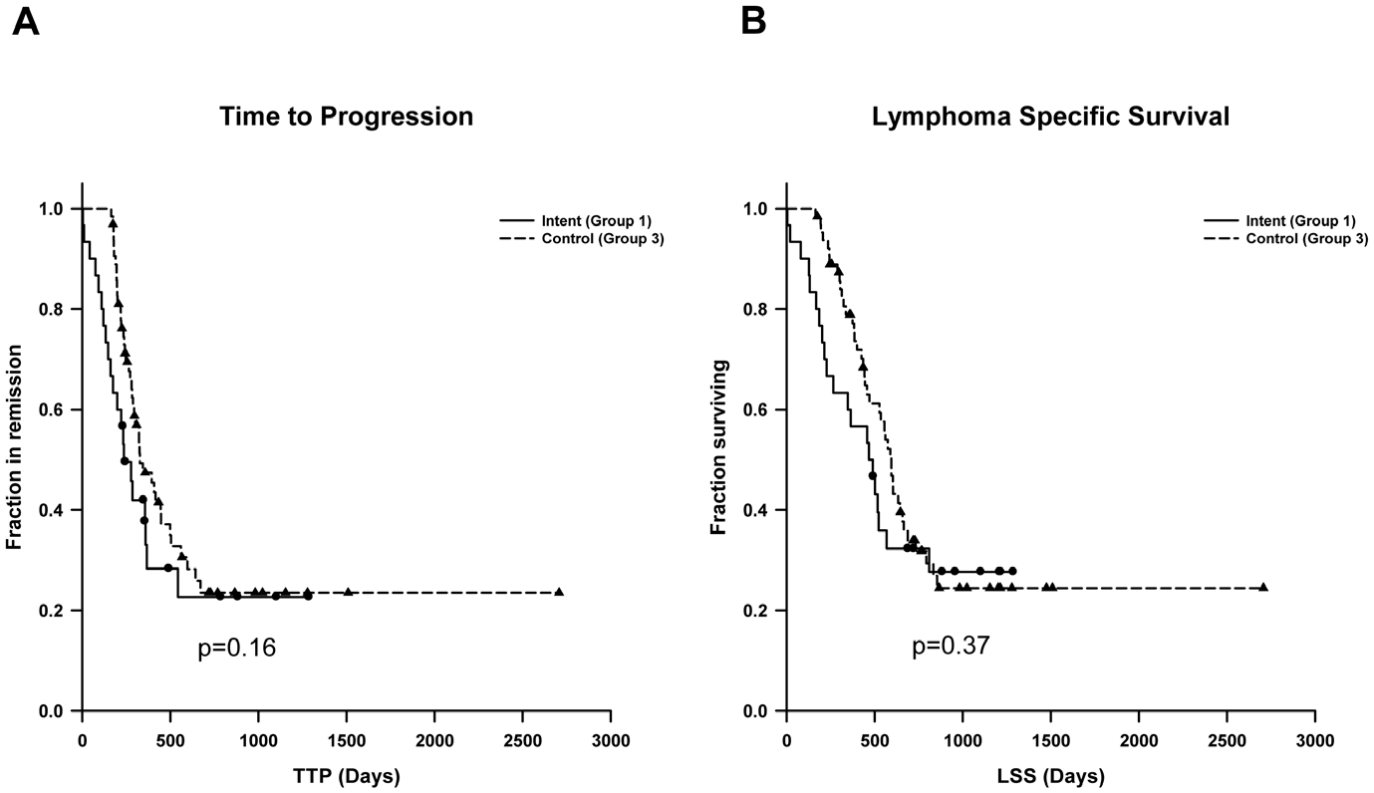
Kaplan Meier estimates comparing intent-to-treat Group 1 to unvaccinated control Group 3 for (A) time-to-progression (TTP) (p = 0.16) and (B) lymphoma-specific survival (LSS) (p = 0.37).

**Figure 2:**
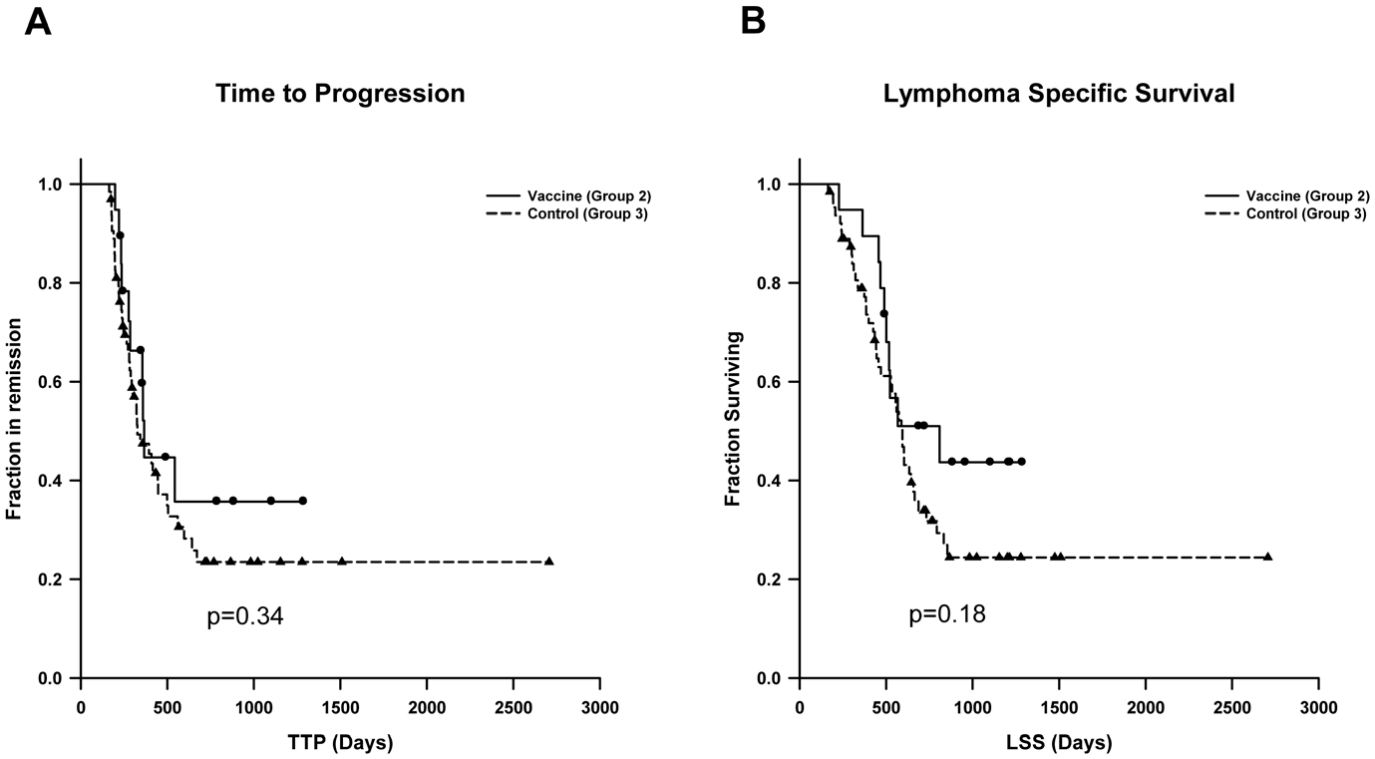
Kaplan Meier estimates comparing vaccinated Group 2 to unvaccinated control Group 3 for (A) TTP (p = 0.34) and (B) LSS (p = 0.18).

Median time to disease progression (TTP) was 236 days (95% confidence intervals [CI], 158–314 days) in Group 1 (intent to treat), 366 days (95% CI, 349–383 days) in Group 2 (vaccinated dogs), and 327 days (95% CI, 223–431) in Group 3 (unvaccinated dogs). There were no statistically significant differences in TTP between Groups 1 and 3 (p = 0.16) or Groups 2 and 3 (p = 0.34). Kaplan Meier curves for TTP are shown in Figs. 1A and 2A. Median lymphoma specific survival (LSS) was 489 days in Group 1 (95% CI, 309–669 days), 809 days in Group 2 (95% CI, 289–1335 days), and 594 days in the Group 3 (95% CI, 536–652 days). Kaplan Meier curves for LSS are shown in Figs. 1B and 2B. Although there was a 36% improvement in the median LSS in the vaccinated Group 2 compared to the unvaccinated control Group 3, the difference was not statistically significant (p = 0.18). Similarly, there was no statistically significant difference in median LLS between Group 1 and Group 3 (p = 0.37). The overall survival rate in Group 2 and Group 3 dogs at 1 year was 89.5% and 74.1%, respectively, and at year 2 was 41.4% and 25.4%, respectively. There was no statistically significant difference in overall survival, indicating that vaccination did not increase mortality.

Fifteen of 19 (79%) vaccinated dogs in Group 2 eventually relapsed, but 4 dogs (21%) did not and achieved a durable (>16 months) first remission after chemotherapy and CD40-B vaccination. One of these four dogs died from unrelated causes at 492 days with no evidence of lymphoma confirmed by necropsy. The other 3 dogs are alive with no evidence of lymphoma at 959, 1103, and 1287 days after the start of chemotherapy (with clinical follow up ongoing). Ten of the vaccinated dogs (52.6%) have died due to lymphoma; six remain alive in durable first or second remission, and 3 were euthanized due to other causes. Two of the three dogs euthanized had hemangiosarcoma, with no evidence of lymphoma, and the other dog was euthanized due to “declining health.” Limited work-up was allowed on this dog, but necropsy found no evidence of lymphoma. The cause of declining health was not identified; however, there was no evidence of autoimmunity and all cell lineages with full maturation were present in the bone marrow. A total of 7 dogs underwent necropsies when they died or were euthanized. With the exception of the 3 previously discussed dogs, all the other dogs (4 dogs) were euthanized due to advanced stage lymphoma and had no evidence of late toxicities from the vaccine. Four of the 64 dogs in the control group (Group 3) were lost to follow-up after restaging, and outcome information was not available. Of the remaining 60 dogs, 46 (76.7%) relapsed and 14 (23.3%) did not. There was no significant difference in relapse rate between Groups 2 and 3 (p = 1.0).

### Salvage therapy and cause of death

Ten of the 15 vaccinated dogs (66.7%) in Group 2 that relapsed were treated with cyclophosphamide, vincristine, and prednisone (COP) as salvage therapy and four of these dogs (40%) achieved durable second clinical remission (>22 months) and did not relapse. The other 5 vaccinated dogs that relapsed received single-agent prednisone as salvage therapy, one of which achieved a durable second remission and did not relapse. Three of these four vaccinated dogs with a chemotherapy-induced durable second remission are still alive with no evidence of lymphoma at 689, 1209, and 1216 days after the start of their initial chemotherapy. The two other dogs (one treated with COP chemotherapy and 1 treated with prednisone alone) were euthanized at 722 and 958 days respectively; as noted above, the first dog had hemangiosarcoma and the second dog was euthanized due to “declining health.” Neither dog had evidence of lymphoma at necropsy. The other ten dogs relapsed during salvage therapy and were euthanized due to their lymphoma.

**Figure 3:**
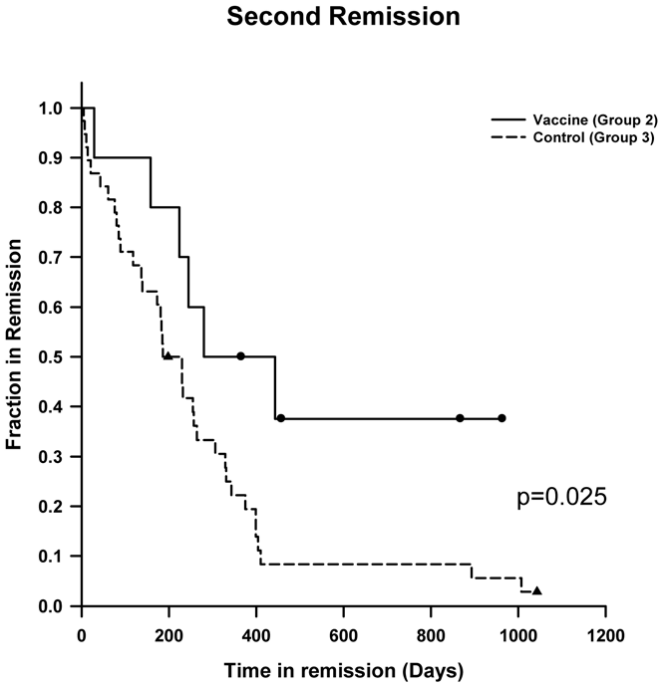
Kaplan Meier estimates for lymphoma-specific survival (LSS) of relapsed dogs who received salvage therapy, demonstrating statistically significant differences between vaccinated Group 2 to unvaccinated control Group 3 (p = 0.038).

In the unvaccinated Group 3, 39 of 46 dogs (84%) that relapsed were treated with salvage chemotherapy and one dog received prednisone alone; however, only 3 (7.7%) of the dogs in Group 3 that received salvage therapy achieved a durable second remission, compared to 40% of vaccinated dogs that achieved a durable second remission to salvage therapy, a difference that was statistically significant (p = 0.025). This difference resulted in a statistically significant improvement in survival after salvage therapy for relapsed Group 2 dogs receiving salvage therapy compared to relapsed Group 3 dogs who received salvage therapy (p = 0.038) (Fig. 3). Importantly, there was no significant difference in the use of salvage therapy between dogs that relapsed in Group 2 (66.7%) and Group 3 (84%), (p = 0.15).

### Immunological assessment

**Figure 4:**
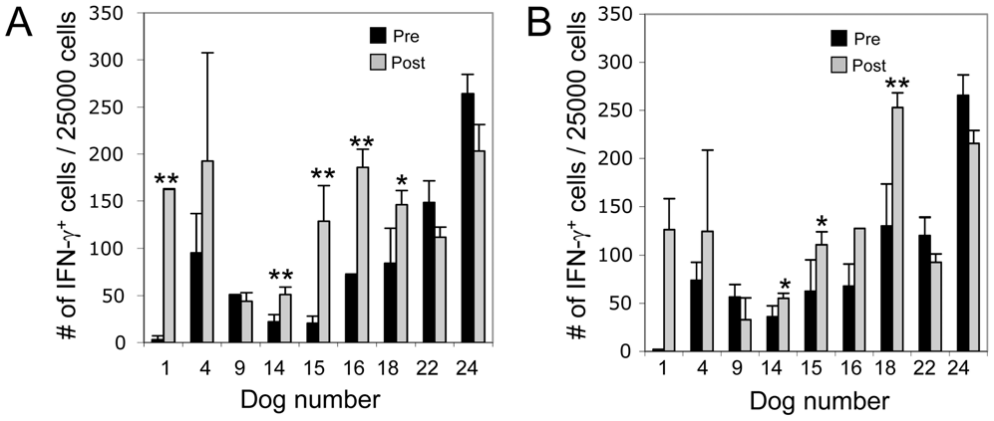
RNA loaded CD40-B cells stimulate antigen-specific IFN-γ responses in PBMCs. PBMCs were obtained from dogs at the time of diagnosis (pre) and 3 weeks post vaccination (post) and antigen-specific immune responses directed against (A) CDV-HA and (B) lymph node tumor antigens were determined by IFN-γ ELISPOT. *p<0.1, **p<0.05.

To determine whether tumor RNA-loaded CD40-B cells induced functional tumor-specific T cell responses *in vivo*, peripheral blood mononuclear cells (PBMC) obtained at the time of diagnosis and 3 weeks after the last vaccination were analyzed for the presence of IFN-γ producing, tumor-specific T cells by ELISPOT. IFN-γ ELISPOT analysis was available for 9 dogs in Group 2 (Fig. 4). A positive IFN-γ response to therapy was defined as >50% increase in the number of cytokine secreting cells after vaccination compared to baseline and with a p value <0.05. Positive immune responses to CDV HA were observed in 4 of 9 vaccinated dogs; a positive IFN-γ immune response to autologous tumor antigen was also observed in 1 dog and a trend towards significance (p<0.1) was observed in another 2 dogs. In 2 other dogs (dog 1 and dog 16) there was >50% increase in the number of cytokine secreting cells in response to autologous tumor antigen after vaccination compared to baseline, but statistical significance could not be assigned due to duplicate rather than triplicate analysis. There was no correlation between immunological response and the average number of CD40-B cells administered to each dog over the course of the three vaccinations. Because only 9 dogs were able to be analyzed (due to insufficient availability of PBMC at all time points), we were unable to detect a statistically significant correlation between immunological response and clinical outcome.

## Discussion

The aim of this study was to determine whether tumor RNA-transfected CD40-activated B cells could safely induce anti-tumor immunity and impact the natural history of spontaneous NHL in dogs after remission induction with chemotherapy. In a clinical trial of privately owned dogs, RNA-loaded autologous CD40-B cells administered intradermally in the setting of minimal disease functioned as APC *in vivo* and stimulated IFN-γ secreting cells. Although our vaccination approach did not impact TTP or LSS when compared to a control group of dogs treated with chemotherapy alone, we observed a statistically significant improvement in the vaccinated group in the rate of durable second remissions as well as subsequent lymphoma-specific survival in relapsed dogs following treatment with salvage therapy. This finding is particularly intriguing because the salvage therapy used included only drugs that had already been used in the initial chemotherapy regimen. It has long been appreciated that the vast majority of dogs with NHL that relapse after initial remission suffer rapid tumor progression and die despite salvage treatments [15]. As an illustration of this, in the chemotherapy (no vaccine) group in this study, durable second remission was achieved in only 7.7% of dogs. In contrast, for dogs that relapsed after receiving chemotherapy plus CD40-B vaccination, 40% of dogs achieved a second, durable remission. Moreover, lymphoma-specific survival was longer in relapsed dogs that had been previously vaccinated than those dogs that had not (with active clinical follow-up ongoing). Although the number of dogs in the vaccinated group is small, the clinical findings in this feasibility trial are encouraging and warrant further testing in a larger, double-blinded, randomized clinical trial. Overall, our clinical and immunological results suggest that cell-based CD40 cancer vaccination synergizes with chemotherapy to improve clinical outcome in this disease.

The vaccine strategy employed in this study builds on our previous preclinical work [10], [12] demonstrating that CD40-B may be used as an alternative to DC in cancer vaccine strategies, with the added benefit of *ex vivo* cell expansion allowing multiple vaccines to be generated from a small volume of peripheral blood. Our results confirm that the use of whole tumor RNA transfected CD40-B cells as an alternative to DC vaccination is both feasible and well tolerated *in vivo*. In this study we used whole tumor RNA as the antigenic payload that supplies both mutated tumor/lymphoma specific antigens and non-mutated self-antigens. *In vitro*, RNA-transfected CD40-B can stimulate cytotoxic T cell responses against tumor antigens that are comparable with responses induced by antigen-loaded DC [10], [16]. Here, we have translated these findings into the clinical arena and show that, similar to antigen-loaded DC, CD40-B can induce immune responses when administered *in vivo*. Importantly, we found that autologous RNA loaded CD40-B could be administered safely to dogs with only occasional and mild injection site reactions noted as acute adverse events. One dog had a single anaphylactic-like reaction to the first vaccination which did not occur following the second or third vaccination. No long-term complications of vaccination including autoimmunity were identified either clinically or at necropsy in any of the dogs. Furthermore, we also evaluated overall survival between the vaccinated group and the control group and found no statistically significant difference, indicating that vaccination did not increase mortality.

Our observations that prior CD40-B vaccination positively impacts the rate of second remission and subsequent survival in relapsed dogs treated with salvage therapy parallels findings that are emerging from recent work in human vaccine trials; namely, prolonged median overall survival [1] or an unexpected high response rate to salvage therapy [17], without necessarily improving progression free survival in human patients vaccinated with antigen-loaded DC. These human studies documented an association between early immune responses to vaccination and unexpected and durable responses to later chemotherapy, and suggest a complex interaction between chemotherapy and immunotherapy that may be therapeutically exploitable. Although the underlying mechanisms behind these observations are not completely understood, it is hypothesized that the use of cytotoxic chemotherapy in patients whose immune systems have been appropriately primed to recognize tumor antigen via cancer vaccination can augment anti-tumor immunity [18]–[19]. Cytotoxic agents such as the anthracyclines may induce “immunogenic” tumor cell death and the release of potent pro-inflammatory molecules that promote DC activation and antigen processing and presentation [20]. Moreover, the cytotoxic effects of salvage therapy may produce an immunological anti-tumor booster effect by breaking down tumor cells and thereby releasing tumor antigens for presentation [21]. Very recent data also suggest that chemotherapy may sensitize tumor targets for killing by specific T cells induced by vaccination [22]. Cytotoxic agents such as cyclophosphamide may also enhance anti-tumor immunity through inhibition of regulatory T cells [23] and by modulating the balance of dendritic cell subsets in lymphoid organs [24]. Thus, in our study, it is possible that salvage therapy served to augment anti-tumor immune responses that had been appropriately primed by prior CD40-B vaccination, leading to durable second remission times and prolonged overall survival.

We used data from dogs treated with the same chemotherapy protocol at the same institution as the control population in the TTP/LSS analyses. Ideally, the control population would have consisted of dogs randomized to receive chemotherapy alone, and the comparisons performed on intent-to-treat basis; however, as many as 40% of dogs presenting to our institution with NHL fail induction chemotherapy and would therefore not be eligible for vaccination or inclusion in the control population. The sample size required to have adequate power for such a study would be quite large and therefore not practical for a feasibility study. As an alternative, we selected dogs with the same disease, treated with the same 20-week chemotherapy regime that led to a complete remission from a larger group of dogs with NHL as an unvaccinated control group. Importantly, by analyzing previously documented negative prognostic factors between groups 2 and 3 (Table 1), we confirmed that there was no statistical difference in these factors between groups 2 and 3. However, due to the small population size of group 2 and the diversity of variables it is possible that differences in negative prognostic factors between the groups may have existed.

Finally, our findings underscore the value of a comparative oncology approach [13], [25] in which privately owned dogs with NHL represent not only a major clinical problem in veterinary medicine but also a spontaneous animal model for the human condition. The testing of novel immune therapies in the high-risk but minimal residual disease setting in dogs is appropriate and ethical, and potentially crucial for the successful development of immunological approaches that would otherwise be suppressed by large tumor burdens. Here, rather than deploying complex therapeutic efforts to address issues of immunosuppression in the tumor microenvironment, we first used standard chemotherapy to massively decrease the tumor burden prior to immune therapy. In the vast majority of phase I clinical trials that evaluate immune therapies in humans, patients have significant tumor burden, advanced disease, and have suffered the immunosuppressive effects of multiple rounds of chemotherapy and other treatments. The study of dogs with spontaneous NHL provides a unique opportunity to evaluate the therapeutic efficacy of immune therapies in the setting of minimal residual disease. The results from this study suggest that synergistic effects between chemotherapy and immunotherapy occur and that the optimal combination of these modalities may lead to significant therapeutic responses. Further studies in privately owned dogs with naturally occurring cancers may provide an opportunity to understand the underlying mechanisms behind this effect and to determine the optimal combination and sequence of chemo-immunotherapy for therapeutic success. This approach may speed the translation of such combinations to human patients.

## Materials and Methods

### Ethics Statement

The study was approved by our university's Institutional Animal Care and Use Committee (Protocol 800900) and the Privately Owned Animal Protocol Committee of the University of Pennsylvania School of Veterinary Medicine (Protocol POAP-197).

### Protocol study population, eligibility criteria and study design

Dogs with newly diagnosed, untreated multicentric NHL that weighed more than 5 kg were screened for eligibility to participate in this study. At the time of diagnosis, complete staging, including CBC, serum chemistry profile, urinalysis, thoracic radiographs, abdominal ultrasound, immunophenotyping, bone marrow aspirates, lymph node aspirates and biopsies, was performed. Eligibility criteria included no serious concurrent disease, less than 30% lymphoblasts on bone marrow aspirates, rare to absent lymphoblasts detected on peripheral blood smear review, and written informed consent from the owner.

Prior to induction chemotherapy a single, peripheral lymph node was surgically excised, and peripheral blood was collected for vaccine generation and baseline immune function studies. Induction chemotherapy consisted of a short, 20-week combination chemotherapy protocol including cyclophosphamide, doxorubicin, vincristine, L-asparaginase, and prednisone, as previously described [14]. The same induction chemotherapy protocol was used for dogs in groups 2 and 3. All dogs underwent complete restaging, including fine needle aspirates and cytological evaluation of a peripheral lymph node, two weeks after completion of the protocol. Only dogs that were confirmed to be in complete remission were eligible for vaccination and only these dogs underwent further blood draws for PBMC collection.

The autologous vaccine consisted of total tumor RNA-loaded CD40-B cells and the control vaccine consisted of CD40-B cells loaded with CDV-HA mRNA. Vaccinations were initiated 3 to 4 weeks after completion of chemotherapy. Vaccinated areas were shaved and aseptically prepared prior to vaccination. Vaccines were given intradermally (tumor RNA CD40-B in one flank; CDV-HA CD40-B in the opposite flank) every 2 to 3 weeks for a total of three vaccinations. Blood for immune studies and toxicity monitoring was collected immediately prior to each vaccination and 3 weeks after the last vaccine was given.

Dogs that failed to achieve remission and therefore did not receive the vaccine and dogs that were vaccinated, but relapsed later were offered standard-of-care salvage therapy according to their owners' preference and veterinarian recommendations. All vaccinated dogs were monitored for relapse through monthly examinations for the first year, every other month for the 2^nd^ year and every 3 months for the third year. Relapses were confirmed by fine needle aspirates in all dogs.

### RNA and mRNA preparation

Manufacturing details for CD40-B preparation, RNA isolation and electroporation have been previously reported [12]. In brief, total RNA was extracted from malignant lymph node tissue taken at the time of diagnosis and prior to chemotherapy using the RNAeasy kit (Qiagen, Valencia, CA). RNA quantity was determined by measuring O.D. at 260 nm and RNA quality was determined by gel electophoresis. RNA was stored at −80°C prior to use. mRNA encoding full-length CDV-HA or GFP was prepared from template plasmids by in vitro transcription as previously described [12]. Transcribed RNA was polyadenylated at the 3′ terminus using the Escherichia coli Poly(A) Polymerase I (E-PAP), treated with DNAse to remove plasmid sequences and purified by acidic phenol/chloroform extraction and RNeasy column separation (Qiagen).

### Vaccine manufacturing

CD40-B were generated from canine peripheral blood mononuclear cells (PBMC) using K562 cells transfected with human CD40L (KtCD40L) as feeder cells as previously described [12]. 5×10^6^ PBMCs were co-cultured with 10^6^ lethally irradiated KtCD40L cells in the presence of recombinant canine IL-4 and cyclosporine. After 5–7 days, B cells were harvested, counted and re-stimulated with irradiated KtCD40L cells in the presence of IL-4 and cyclosporine. Cells were stimulated with KtCD40L cells three times. Five days following the last stimulation CD40-B harvested, washed twice in PBS and resuspended at 2×10^6^ cells per 100 ul of Nucleofector B solution (Amaxa, Cologne, Germany). Up to 5×10^6^ cells were electroporated with either 10 ug autologous tumor RNA or 2 ug CDV mRNA using the Amaxa Nuceofector device (pulse program U08). The electroporator was designed and manufactured according to GMP standards set by the FDA and is approved for use in human vaccine manufacturing. Following electroporation, cells were washed once in B cell media and then twice in PBS. Electroporated cells were resuspended in 300 ul of PBS and administered intradermally over the right flank for tumor RNA-loaded CD40-B cells and over the left flank for CDV-loaded CD40-B cells.

### Vaccine preparation and generation

CD40-B cells and tumor RNA were generated from all dogs although the number of CD40-B cells produced varied per dog. Dogs received the same number of RNA-loaded CD40-B cells for CDV and tumor vaccination and the majority of dogs were vaccinated with CD40-B cells in the target range of 1×10^6^ to 5×10^6^ cells. In three dogs, B cells did not expand well using the KtCD40L culture system and CD40-B cell vaccine doses were less than 10^6^ cells per vaccine. Following electroporation both CDV and tumor RNA loaded CD40-B cells for each dog were submitted for aerobic and anaerobic bacterial culture and fungal culture to determine the presence of microbial contaminants. Of the 114 vaccines generated, two (from the same batch of CD40-B cells) tested positive for non-fermenting gram negative bacilli. Fungal cultures from all vaccines were negative. All dogs except for one, received vaccines as scheduled every three weeks as per protocol. In one case only, the third vaccination was delayed by one week as a result of low surface expression of CD40L on feeder cells and the subsequent failure to culture B cells.

### Clinical control group

Dogs with multicentric NHL that were treated at the same institution with the same 20-week combination chemotherapy protocol and that achieved complete clinical remission confirmed by restaging were used as a control population for comparison in the outcome analysis. Information regarding signalment, stage, immunophenotype (when available), TTP, LSS, use of salvage therapy, and cause of death were collected and compared to the vaccine group.

### IFN-gamma ELISPOT immune analysis

To amplify antigen-specific T cell responses, cryopreserved PBMC were thawed, and stimulated with autologous CD40-B electroporated with either 2 ug CDV mRNA or 10 ug autologous tumor RNA. 3×10^6^ PBMC were co-cultured with 5×10^5^ RNA-loaded CD40-B cells in 24-well plates as previously described [12]. Cultured were supplemented with 500 U/ml recombinant canine IL-4 and 10 ng/ml recombinant human IL-7 (Sigma, St. Louis, MO, USA) on day 0 and 20 U/ml recombinant human IL-2 (Chiron, Emeryville, CA, USA) on days 1 and 4. After 7 days, cells were harvested and used for ELISPOT (2.5×10^4^ T cells per well) as previously described [12]. All samples were performed in triplicate; for cases in which cell numbers were limiting, samples were performed in duplicate. Targets were autologous PBMC stimulated with phytohemagglutinin (PHA, 5 ug/ml) and IL-2 for 5 days, electroporated with either CDV mRNA or total autologous tumor RNA. Immunospot plates were pre-coated with anti-canine IFN-γ monoclonal antibody (R&D Systems) and incubated at 37°C overnight. Plates were developed according to the manufacturer's instructions and spots were counted using a Prior ProScan analyzer and Image Pro Plus software (Hitech Instruments, Edgemont, PA, USA).

### Biostatistics

Continuous data were expressed as means (+/− SD) and categorical data as frequencies and percentages. To assess differences between Groups 1, 2, and 3, Student's t-test was used for continuous variables (e.g. age, body weight) and Chi-square or Fisher's exact test was used for categorical or binominal variables. Time to progression (TTP) and lymphoma specific survival (LSS) were the endpoints evaluated in the outcome analysis, measured from the first day of chemotherapy to the clinical event in all groups. Dogs that were lost to follow-up and dogs that died due to other causes than lymphoma and lymphoma treatment were censored at the last date of known status or when they died from other causes, respectively. The Kaplan Meier product limit method was used to estimate TTP and LSS for all 3 groups. Censored data was entered into the Kaplan Meier model and handled as incomplete data for the computations [26]. The log rank test was used to test for differences between the groups. Significance was set at p<.05. ELISPOT results pre and post vaccination were compared using Student's t-tests. Significance was set at **p<0.05, and results that approached significance *p<0.1 were also noted. In cases for which samples could only be performed in duplicate, p values could not be calculated.
